# Dichlorido(5,5′-dimethyl-2,2′-bipyridine-κ^2^ *N*,*N*′)zinc(II)

**DOI:** 10.1107/S1600536808027104

**Published:** 2008-08-30

**Authors:** Aida Khalighi, Roya Ahmadi, Vahid Amani, Hamid Reza Khavasi

**Affiliations:** a Islamic Azad University, Shahr-e-Rey Branch, Tehran, Iran; b Department of Chemistry, Shahid Beheshti University, Tehran 1983963113, Iran

## Abstract

The asymmetric unit of the title compound, [ZnCl_2_(C_12_H_12_N_2_)], contains two independent mol­ecules. The Zn^II^ atoms are four-coordinated in distorted tetra­hedral configurations by two N atoms from 5,5′-dimethyl-2,2′-bipyridine and two terminal Cl atoms. In the crystal structure, inter­molecular C—H⋯Cl hydrogen bonds link the mol­ecules. There are C—H⋯π contacts between the methyl groups and the pyridine and five-membered rings containing Zn^II^ atoms; π–π contacts also exist between the pyridine rings [centroid–centroid distances = 3.665 (5) and 3.674 (5) Å].

## Related literature

For related literature, see: Gruia *et al.* (2007 ▶); Khan & Tuck (1984 ▶); Khavasi *et al.* (2008 ▶); Kozhevnikov *et al.* (2006 ▶); Liu *et al.* (2004 ▶); Lundberg (1966 ▶); Preston & Kennard (1969 ▶); Qin *et al.* (1999 ▶); Reimann *et al.* (1966 ▶); Steffen & Palenik (1976 ▶, 1977 ▶).
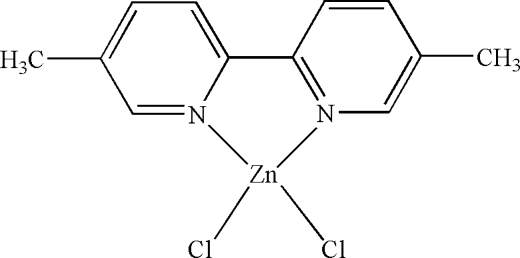


## Experimental

### 

#### Crystal data

[ZnCl_2_(C_12_H_12_N_2_)]
*M* *_r_* = 320.53
Orthorhombic, 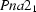

*a* = 16.267 (2) Å
*b* = 11.1704 (16) Å
*c* = 14.9328 (14) Å
*V* = 2713.4 (6) Å^3^
*Z* = 8
Mo *K*α radiation
μ = 2.18 mm^−1^
*T* = 298 (2) K
0.28 × 0.20 × 0.07 mm

#### Data collection

Bruker SMART CCD area-detector diffractometer
Absorption correction: multi-scan (*SADABS*; Sheldrick, 1998 ▶) *T*_min_ = 0.612, *T*_max_ = 0.860
14309 measured reflections
7167 independent reflections
4463 reflections with *I* > 2σ(*I*)
*R*_int_ = 0.066

#### Refinement

*R*[*F* ^2^ > 2σ(*F* ^2^)] = 0.066
*wR*(*F* ^2^) = 0.150
*S* = 1.07
7167 reflections
307 parameters
1 restraint
H-atom parameters constrained
Δρ_max_ = 0.29 e Å^−3^
Δρ_min_ = −0.30 e Å^−3^
Absolute structure: Flack (1983 ▶), 3320 Friedel pairs
Flack parameter: 0.07 (3)

### 

Data collection: *SMART* (Bruker, 1998 ▶); cell refinement: *SAINT* (Bruker, 1998 ▶); data reduction: *SAINT*; program(s) used to solve structure: *SHELXTL* (Sheldrick, 2008 ▶); program(s) used to refine structure: *SHELXTL*; molecular graphics: *ORTEP-3 for Windows* (Farrugia, 1997 ▶); software used to prepare material for publication: *WinGX* (Farrugia, 1999 ▶).

## Supplementary Material

Crystal structure: contains datablocks I, global. DOI: 10.1107/S1600536808027104/hk2517sup1.cif

Structure factors: contains datablocks I. DOI: 10.1107/S1600536808027104/hk2517Isup2.hkl

Additional supplementary materials:  crystallographic information; 3D view; checkCIF report

## Figures and Tables

**Table:** 

Zn1—Cl1	2.206 (2)
Zn1—Cl2	2.215 (2)
Zn2—Cl3	2.211 (2)
Zn2—Cl4	2.207 (3)
Zn1—N1	2.058 (6)
Zn1—N2	2.057 (6)
Zn2—N3	2.063 (6)
Zn2—N4	2.066 (6)

**Table:** 

N1—Zn1—N2	80.5 (2)
N1—Zn1—Cl1	112.2 (2)
N1—Zn1—Cl2	117.23 (18)
N2—Zn1—Cl1	115.64 (18)
N2—Zn1—Cl2	111.4 (2)
Cl1—Zn1—Cl2	115.28 (10)
N3—Zn2—N4	79.7 (3)
N3—Zn2—Cl3	112.09 (18)
N3—Zn2—Cl4	114.47 (17)
N4—Zn2—Cl4	112.8 (2)
N4—Zn2—Cl3	115.01 (18)
Cl4—Zn2—Cl3	117.19 (9)

**Table 2:** Hydrogen-bond geometry (Å, °)

*D*—H⋯*A*	*D*—H	H⋯*A*	*D*⋯*A*	*D*—H⋯*A*
C1—H1⋯Cl3^i^	0.93	2.82	3.516 (8)	132
C16—H16⋯Cl3^ii^	0.93	2.83	3.638 (10)	146
C3—H3*A*⋯*Cg*5	0.96	3.10	3.719 (6)	124
C11—H11*A*⋯*Cg*2^iii^	0.96	2.83	3.688 (5)	150
C15—H15*C*⋯*Cg*1^iv^	0.96	2.84	3.704 (6)	150
C23—H23*C*⋯*Cg*4	0.96	3.11	3.690 (6)	120

Symmetry codes: (i) 
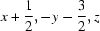
; (ii) 
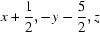
; (iii) 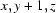; (iv) 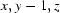. *Cg*1, *Cg*2, *Cg*4 and *Cg*5 are the centroids of atoms Zn1/N1/C6/C7/N2, N1/C1/C2/C4–C6, Zn2/N3/C18/C19/N4 and N3/C13/C14/C16–C18, respectively.

**Fig. 1.**
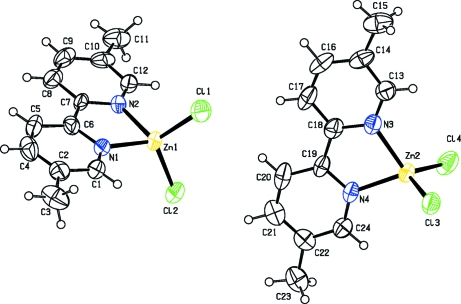
The molecular structure of the title molecule, with the atom-numbering scheme. Displacement ellipsoids are drawn at the 40% probability level.

**Fig. 2.**
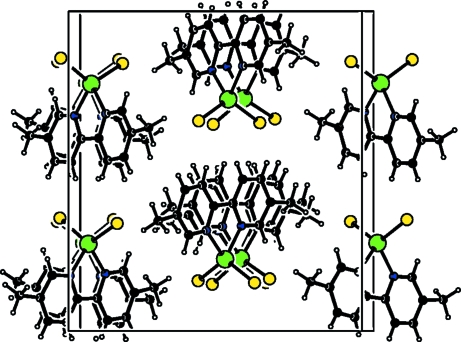
A packing diagram of the title compound.

## supplementary crystallographic information

### Comment

There are several Zn^II^ complexes, with formula, [ZnCl_2_(N—N)], such as [ZnCl_2_(bipy)], (II), (Khan & Tuck, 1984), [ZnCl_2_(biim)], (III), (Gruia *et al.*, 2007), [ZnCl_2_(phbipy)], (IV), (Kozhevnikov *et al.*, 2006), [ZnCl_2_(phen)], (V), (Reimann *et al.*, 1966), [ZnCl_2_(dmphen)], (VI), (Preston & Kennard, 1969), [ZnCl_2_(dpdmbip)], (VII), (Liu *et al.*, 2004) and [ZnCl_2_(dm4bt)], (VIII), (Khavasi *et al.*, 2008) [where bipy is 2,2'-bipyridine, biim is 2,2'-biimidazole, phbipy is 5-phenyl-2,2'-bi- pyridine, phen is 1,10-phenanthroline, dmphen is 2,9-dimethyl-1,10-phenanthro- line, dpdmbip is 4,4'-diphenyl-6,6'-dimethyl-2,2'-bipyrimidine and dm4bt is 2,2'-dimethyl-4,4'-bithiazole] have been synthesized and characterized by single-crystal X-ray diffraction methods. There are also several Zn^II^ complexes, with formula, [ZnCl_2_*L*_2_], such as [ZnCl_2_(py)_2_], (IX), (Steffen & Palenik, 1976), [ZnCl_2_(4-cypy)_2_], (*X*), (Steffen & Palenik, 1977), [ZnCl_2_(2-ampy)_2_], (XI), (Qin *et al.*, 1999) and [ZnCl_2_(im)_2_], (XII), (Lundberg, 1966), [where py is pyridine, 4-cypy is 4-cyanopyridine, 2-ampy is 2-aminopyridine and im is imidazole] have been synthesized and characterized by single-crystal X-ray diffraction methods. We report herein the synthesis and crystal structure of the title compound, (I).

The asymmetric unit of (I), (Fig. 1), contains two independent molecules. The Zn^II^ atoms are four-coordinated in distorted tetrahedral configurations (Table 1) by two N atoms from 5,5'-dimethyl-2,2'-bipyridine and two terminal Cl. The Zn-Cl and Zn-N bond lengths and angles (Table 1) are within normal ranges, as in (II), (V) and (VIII).

In the crystal structure, intermolecular C-H···Cl hydrogen bonds (Table 2) link the molecules, in which they may be effective in the stabilization of the structure. There also exist C—H···π contacts (Table 1) between the methyl groups and pyridine, (Zn1/N1/N2/C6/C7) and (Zn2/N3/N4/C18/C19) rings. The π—π contacts between the pyridine rings, Cg3···Cg6^i^ and Cg4···Cg5^ii^ [symmetry codes: (i) x, y, z; (ii) x, 1 + y, z, where Cg3, Cg4, Cg5 and Cg6 are centroids of the rings (N1/C1/C2/C4-C6), (N2/C7-C10/C12), (N3/C13/C14/C16-C18) and (N4/C19-C22/C24), respectively] further stabilize the structure, with centroid-centroid distances of 3.665 (5) and 3.674 (5) Å, respectively.

### Experimental

For the preparation of the title compound, a solution of 5,5'-dimethyl-2,2' -bipyridine (0.25 g, 1.33 mmol) in methanol (100 ml) was added to a solution of ZnCl_2_ (0.18 g, 1.33 mmol) in methanol (100 ml) and the resulting colorless solution was stirred for 5 min at room temperature, and then left to evaporate slowly at room temperature. After one week, colorless block crystals of the title compound were isolated (yield; 0.32 g, 73.4%, m.p. < 573 K).

### Refinement

H atoms were positioned geometrically, with C-H = 0.93 and 0.96 Å for aromatic and methyl H, respectively, and constrained to ride on their parent atoms with U_iso_(H) = 1.2U_eq_(C).

### Crystal data

**Table:**

[ZnCl_2_(C_12_H_12_N_2_)]	*F*_000_ = 1296
*M**_r_* = 320.53	*D*_x_ = 1.569 Mg m^−^^3^
Orthorhombic, *P**n**a*2_1_	Mo *K*α radiation λ = 0.71073 Å
Hall symbol: P 2c -2n	Cell parameters from 2610 reflections
*a* = 16.267 (2) Å	θ = 2.2–29.3º
*b* = 11.1704 (16) Å	µ = 2.18 mm^−^^1^
*c* = 14.9328 (14) Å	*T* = 298 (2) K
*V* = 2713.4 (6) Å^3^	Block, colorless
*Z* = 8	0.28 × 0.20 × 0.07 mm

### Data collection

**Table:**

Bruker SMART CCD area-detector diffractometer	7167 independent reflections
Radiation source: fine-focus sealed tube	4463 reflections with *I* > 2σ(*I*)
Monochromator: graphite	*R*_int_ = 0.066
*T* = 298(2) K	θ_max_ = 29.3º
φ and ω scans	θ_min_ = 2.2º
Absorption correction: multi-scan(SADABS; Sheldrick, 1998)	*h* = −22→13
*T*_min_ = 0.612, *T*_max_ = 0.860	*k* = −13→15
14309 measured reflections	*l* = −20→20

### Refinement

**Table:**

Refinement on *F*^2^	Hydrogen site location: inferred from neighbouring sites
Least-squares matrix: full	H-atom parameters constrained
*R*[*F*^2^ > 2σ(*F*^2^)] = 0.066	*w* = 1/[σ^2^(*F*_o_^2^) + (0.0545*P*)^2^ + 2.0209*P*] where *P* = (*F*_o_^2^ + 2*F*_c_^2^)/3
*wR*(*F*^2^) = 0.150	(Δ/σ)_max_ = 0.045
*S* = 1.08	Δρ_max_ = 0.29 e Å^−^^3^
7167 reflections	Δρ_min_ = −0.30 e Å^−^^3^
307 parameters	Extinction correction: none
1 restraint	Absolute structure: Flack (1983), 3320 Friedel pairs
Primary atom site location: structure-invariant direct methods	Flack parameter: 0.07 (3)
Secondary atom site location: difference Fourier map	

### Special details

**Table:**

Geometry. All e.s.d.'s (except the e.s.d. in the dihedral angle between two l.s. planes) are estimated using the full covariance matrix. The cell e.s.d.'s are taken into account individually in the estimation of e.s.d.'s in distances, angles and torsion angles; correlations between e.s.d.'s in cell parameters are only used when they are defined by crystal symmetry. An approximate (isotropic) treatment of cell e.s.d.'s is used for estimating e.s.d.'s involving l.s. planes.
Refinement. Refinement of *F*^2^ against ALL reflections. The weighted *R*-factor *wR* and goodness of fit *S* are based on *F*^2^, conventional *R*-factors *R* are based on *F*, with *F* set to zero for negative *F*^2^. The threshold expression of *F*^2^ > σ(*F*^2^) is used only for calculating *R*-factors(gt) *etc*. and is not relevant to the choice of reflections for refinement. *R*-factors based on *F*^2^ are statistically about twice as large as those based on *F*, and *R*- factors based on ALL data will be even larger.

### Fractional atomic coordinates and isotropic or equivalent isotropic displacement parameters (Å^2^)

**Table:**

	*x*	*y*	*z*	*U*_iso_*/*U*_eq_	
Zn1	−0.27268 (5)	−0.87606 (8)	−0.43784 (5)	0.0530 (4)	
Zn2	−0.77530 (5)	−1.11325 (7)	−0.47118 (5)	0.0521 (3)	
Cl1	−0.33658 (15)	−0.9839 (2)	−0.33543 (19)	0.0872 (8)	
Cl2	−0.35350 (12)	−0.7899 (2)	−0.53825 (16)	0.0671 (6)	
Cl3	−0.84859 (13)	−1.0208 (2)	−0.36748 (16)	0.0644 (6)	
Cl4	−0.84271 (14)	−1.2264 (2)	−0.56774 (19)	0.0823 (8)	
N1	−0.1798 (4)	−0.7734 (5)	−0.3840 (4)	0.0508 (16)	
N2	−0.1730 (4)	−0.9564 (5)	−0.4975 (4)	0.0508 (15)	
N3	−0.6710 (4)	−1.1912 (5)	−0.4188 (4)	0.0521 (14)	
N4	−0.6855 (4)	−1.0095 (6)	−0.5312 (5)	0.0503 (15)	
C1	−0.1882 (5)	−0.6828 (7)	−0.3260 (5)	0.0541 (18)	
H1	−0.2408	−0.6608	−0.3081	0.065*	
C2	−0.1206 (6)	−0.6198 (7)	−0.2909 (6)	0.060 (2)	
C3	−0.1361 (7)	−0.5168 (8)	−0.2285 (6)	0.089 (3)	
H3A	−0.1689	−0.4576	−0.2584	0.107*	
H3B	−0.0846	−0.4819	−0.2108	0.107*	
H3C	−0.1648	−0.5449	−0.1764	0.107*	
C4	−0.0441 (6)	−0.6540 (9)	−0.3175 (6)	0.071 (2)	
H4	0.0018	−0.6146	−0.2949	0.085*	
C5	−0.0340 (5)	−0.7457 (9)	−0.3773 (6)	0.062 (2)	
H5	0.0183	−0.7674	−0.3963	0.075*	
C6	−0.1031 (5)	−0.8063 (7)	−0.4095 (5)	0.0524 (19)	
C7	−0.1007 (4)	−0.9082 (7)	−0.4741 (6)	0.0459 (17)	
C8	−0.0275 (5)	−0.9510 (8)	−0.5090 (5)	0.061 (2)	
H8	0.0226	−0.9168	−0.4930	0.073*	
C9	−0.0309 (5)	−1.0467 (9)	−0.5686 (6)	0.069 (3)	
H9	0.0177	−1.0773	−0.5921	0.082*	
C10	−0.1045 (7)	−1.0967 (8)	−0.5934 (6)	0.062 (3)	
C11	−0.1117 (7)	−1.1986 (8)	−0.6598 (6)	0.082 (3)	
H11A	−0.1368	−1.2664	−0.6311	0.098*	
H11B	−0.0579	−1.2202	−0.6807	0.098*	
H11C	−0.1449	−1.1739	−0.7096	0.098*	
C12	−0.1746 (5)	−1.0486 (7)	−0.5547 (6)	0.057 (2)	
H12	−0.2252	−1.0820	−0.5694	0.068*	
C13	−0.6682 (5)	−1.2826 (7)	−0.3606 (6)	0.059 (2)	
H13	−0.7178	−1.3166	−0.3429	0.071*	
C14	−0.5966 (6)	−1.3302 (9)	−0.3250 (6)	0.067 (2)	
C15	−0.5985 (7)	−1.4306 (9)	−0.2602 (6)	0.089 (3)	
H15A	−0.6285	−1.4068	−0.2078	0.107*	
H15B	−0.5434	−1.4516	−0.2438	0.107*	
H15C	−0.6251	−1.4985	−0.2871	0.107*	
C16	−0.5243 (6)	−1.2783 (9)	−0.3560 (7)	0.074 (3)	
H16	−0.4741	−1.3087	−0.3370	0.089*	
C17	−0.5255 (5)	−1.1830 (9)	−0.4141 (6)	0.074 (2)	
H17	−0.4765	−1.1473	−0.4322	0.089*	
C18	−0.6005 (5)	−1.1398 (8)	−0.4458 (7)	0.0552 (19)	
C19	−0.6087 (4)	−1.0390 (7)	−0.5074 (5)	0.0501 (17)	
C20	−0.5433 (5)	−0.9743 (9)	−0.5441 (7)	0.072 (3)	
H20	−0.4894	−0.9962	−0.5310	0.086*	
C21	−0.5580 (6)	−0.8791 (8)	−0.5990 (5)	0.069 (2)	
H21	−0.5140	−0.8339	−0.6199	0.083*	
C22	−0.6369 (6)	−0.8486 (7)	−0.6240 (5)	0.0578 (19)	
C23	−0.6548 (6)	−0.7477 (8)	−0.6847 (6)	0.080 (3)	
H23A	−0.6848	−0.7765	−0.7357	0.096*	
H23B	−0.6042	−0.7120	−0.7042	0.096*	
H23C	−0.6871	−0.6891	−0.6536	0.096*	
C24	−0.6992 (5)	−0.9174 (7)	−0.5874 (5)	0.0572 (18)	
H24	−0.7533	−0.8990	−0.6023	0.069*	

### Atomic displacement parameters (Å^2^)

**Table:**

	*U*^11^	*U*^22^	*U*^33^	*U*^12^	*U*^13^	*U*^23^
Zn1	0.0310 (4)	0.0557 (5)	0.0721 (10)	−0.0013 (4)	0.0036 (4)	0.0035 (4)
Zn2	0.0336 (4)	0.0550 (5)	0.0678 (9)	0.0005 (4)	−0.0026 (4)	−0.0006 (4)
Cl1	0.0615 (14)	0.0871 (16)	0.113 (2)	0.0035 (12)	0.0292 (13)	0.0328 (14)
Cl2	0.0426 (10)	0.0783 (13)	0.0805 (15)	0.0030 (9)	−0.0096 (9)	0.0048 (11)
Cl3	0.0490 (11)	0.0682 (12)	0.0759 (14)	−0.0025 (9)	0.0060 (9)	−0.0081 (10)
Cl4	0.0550 (13)	0.0829 (15)	0.109 (2)	0.0089 (11)	−0.0215 (12)	−0.0355 (14)
N1	0.038 (3)	0.049 (3)	0.065 (4)	−0.002 (3)	−0.007 (3)	0.012 (3)
N2	0.037 (3)	0.047 (3)	0.069 (4)	−0.003 (3)	0.011 (3)	0.009 (3)
N3	0.041 (3)	0.057 (3)	0.058 (4)	−0.001 (3)	−0.001 (3)	−0.011 (3)
N4	0.034 (3)	0.063 (4)	0.055 (4)	−0.002 (3)	0.003 (3)	−0.008 (3)
C1	0.045 (4)	0.057 (4)	0.061 (4)	−0.002 (3)	−0.008 (3)	0.002 (3)
C2	0.065 (5)	0.062 (5)	0.053 (5)	−0.006 (4)	−0.016 (4)	0.013 (4)
C3	0.135 (10)	0.068 (6)	0.066 (6)	−0.018 (6)	−0.023 (6)	0.001 (4)
C4	0.067 (6)	0.080 (6)	0.066 (5)	−0.028 (5)	−0.016 (4)	0.006 (5)
C5	0.035 (4)	0.088 (6)	0.063 (5)	−0.004 (4)	−0.002 (3)	0.010 (5)
C6	0.046 (4)	0.058 (4)	0.054 (4)	−0.010 (3)	−0.005 (3)	0.021 (3)
C7	0.027 (3)	0.056 (4)	0.055 (5)	0.003 (3)	0.007 (3)	0.017 (4)
C8	0.046 (4)	0.073 (5)	0.065 (5)	0.012 (4)	0.008 (3)	0.014 (4)
C9	0.051 (5)	0.093 (7)	0.062 (5)	0.025 (5)	0.021 (4)	0.027 (5)
C10	0.081 (7)	0.057 (4)	0.048 (5)	0.023 (5)	0.013 (4)	0.022 (4)
C11	0.102 (8)	0.073 (6)	0.071 (6)	0.026 (5)	0.017 (5)	0.005 (5)
C12	0.041 (4)	0.059 (5)	0.071 (5)	−0.002 (3)	0.000 (3)	0.017 (4)
C13	0.058 (5)	0.056 (5)	0.064 (5)	0.001 (4)	−0.009 (4)	−0.008 (4)
C14	0.069 (6)	0.070 (5)	0.061 (5)	0.029 (5)	−0.013 (4)	−0.019 (4)
C15	0.112 (9)	0.082 (7)	0.073 (6)	0.028 (6)	−0.029 (6)	−0.006 (5)
C16	0.052 (5)	0.093 (7)	0.078 (6)	0.032 (5)	−0.014 (4)	−0.013 (5)
C17	0.037 (4)	0.098 (7)	0.089 (6)	0.006 (4)	−0.012 (4)	−0.016 (5)
C18	0.048 (4)	0.059 (4)	0.058 (5)	0.008 (4)	0.010 (4)	−0.020 (4)
C19	0.033 (3)	0.068 (4)	0.049 (4)	0.001 (3)	0.002 (3)	−0.018 (3)
C20	0.039 (4)	0.098 (7)	0.078 (6)	−0.011 (4)	0.005 (4)	−0.032 (5)
C21	0.078 (6)	0.076 (6)	0.053 (5)	−0.021 (5)	0.005 (4)	−0.006 (4)
C22	0.077 (6)	0.054 (4)	0.042 (4)	−0.005 (4)	0.008 (3)	−0.008 (3)
C23	0.093 (7)	0.076 (6)	0.071 (6)	−0.018 (5)	0.013 (5)	−0.007 (5)
C24	0.050 (4)	0.066 (5)	0.055 (4)	0.005 (4)	0.002 (3)	−0.002 (4)

### Geometric parameters (Å, °)

**Table:**

Zn1—Cl1	2.206 (2)	C11—H11B	0.9600
Zn1—Cl2	2.215 (2)	C11—H11C	0.9600
Zn2—Cl3	2.211 (2)	C12—N2	1.338 (10)
Zn2—Cl4	2.207 (3)	C12—H12	0.9300
Zn1—N1	2.058 (6)	C13—N3	1.341 (10)
Zn1—N2	2.057 (6)	C13—C14	1.386 (11)
Zn2—N3	2.063 (6)	C13—H13	0.9300
Zn2—N4	2.066 (6)	C14—C16	1.389 (13)
C1—N1	1.339 (10)	C14—C15	1.482 (13)
C1—C2	1.406 (12)	C15—H15A	0.9600
C1—H1	0.9300	C15—H15B	0.9600
C2—C4	1.362 (14)	C15—H15C	0.9600
C2—C3	1.502 (12)	C16—C17	1.374 (13)
C3—H3A	0.9600	C16—H16	0.9300
C3—H3B	0.9600	C17—C18	1.397 (11)
C3—H3C	0.9600	C17—H17	0.9300
C4—C5	1.369 (13)	C18—N3	1.343 (10)
C4—H4	0.9300	C18—C19	1.460 (12)
C5—C6	1.396 (11)	C19—N4	1.341 (9)
C5—H5	0.9300	C19—C20	1.397 (11)
C6—N1	1.357 (10)	C20—C21	1.364 (13)
C6—C7	1.491 (11)	C20—H20	0.9300
C7—N2	1.339 (9)	C21—C22	1.379 (14)
C7—C8	1.387 (10)	C21—H21	0.9300
C8—C9	1.392 (12)	C22—C24	1.384 (12)
C8—H8	0.9300	C22—C23	1.476 (12)
C9—C10	1.372 (14)	C23—H23A	0.9600
C9—H9	0.9300	C23—H23B	0.9600
C10—C12	1.386 (13)	C23—H23C	0.9600
C10—C11	1.514 (13)	C24—N4	1.348 (10)
C11—H11A	0.9600	C24—H24	0.9300
			
N1—Zn1—N2	80.5 (2)	N3—C13—C14	124.8 (8)
N1—Zn1—Cl1	112.2 (2)	N3—C13—H13	117.6
N1—Zn1—Cl2	117.23 (18)	C14—C13—H13	117.6
N2—Zn1—Cl1	115.64 (18)	C13—C14—C16	115.0 (9)
N2—Zn1—Cl2	111.4 (2)	C13—C14—C15	121.6 (9)
Cl1—Zn1—Cl2	115.28 (10)	C16—C14—C15	123.4 (9)
N3—Zn2—N4	79.7 (3)	C14—C15—H15A	109.4
N3—Zn2—Cl3	112.09 (18)	C14—C15—H15B	109.5
N3—Zn2—Cl4	114.47 (17)	H15A—C15—H15B	109.5
N4—Zn2—Cl4	112.8 (2)	C14—C15—H15C	109.5
N4—Zn2—Cl3	115.01 (18)	H15A—C15—H15C	109.5
Cl4—Zn2—Cl3	117.19 (9)	H15B—C15—H15C	109.5
N1—C1—C2	122.6 (8)	C17—C16—C14	121.4 (8)
N1—C1—H1	118.7	C17—C16—H16	119.3
C2—C1—H1	118.7	C14—C16—H16	119.3
C4—C2—C1	117.7 (8)	C16—C17—C18	119.6 (9)
C4—C2—C3	123.4 (9)	C16—C17—H17	120.2
C1—C2—C3	118.9 (9)	C18—C17—H17	120.2
C2—C3—H3A	109.5	N3—C18—C17	119.7 (9)
C2—C3—H3B	109.5	N3—C18—C19	116.2 (7)
H3A—C3—H3B	109.5	C17—C18—C19	124.1 (8)
C2—C3—H3C	109.5	N4—C19—C20	118.6 (8)
H3A—C3—H3C	109.5	N4—C19—C18	116.2 (7)
H3B—C3—H3C	109.5	C20—C19—C18	125.2 (8)
C2—C4—C5	120.8 (8)	C21—C20—C19	120.3 (9)
C2—C4—H4	119.6	C21—C20—H20	119.9
C5—C4—H4	119.6	C19—C20—H20	119.9
C4—C5—C6	119.4 (8)	C20—C21—C22	121.4 (9)
C4—C5—H5	120.3	C20—C21—H21	119.3
C6—C5—H5	120.3	C22—C21—H21	119.3
N1—C6—C5	120.8 (8)	C21—C22—C24	115.9 (8)
N1—C6—C7	114.4 (7)	C21—C22—C23	122.7 (9)
C5—C6—C7	124.8 (8)	C24—C22—C23	121.3 (9)
N2—C7—C8	121.1 (8)	C22—C23—H23A	109.5
N2—C7—C6	116.9 (7)	C22—C23—H23B	109.5
C8—C7—C6	122.0 (8)	H23A—C23—H23B	109.5
C7—C8—C9	118.2 (8)	C22—C23—H23C	109.5
C7—C8—H8	120.9	H23A—C23—H23C	109.5
C9—C8—H8	120.9	H23B—C23—H23C	109.5
C10—C9—C8	121.3 (8)	N4—C24—C22	123.2 (8)
C10—C9—H9	119.4	N4—C24—H24	118.4
C8—C9—H9	119.4	C22—C24—H24	118.4
C9—C10—C12	116.6 (9)	C1—N1—C6	118.8 (7)
C9—C10—C11	123.3 (9)	C1—N1—Zn1	126.7 (5)
C12—C10—C11	120.2 (10)	C6—N1—Zn1	114.5 (5)
C10—C11—H11A	109.4	C12—N2—C7	119.4 (7)
C10—C11—H11B	109.5	C12—N2—Zn1	126.8 (5)
H11A—C11—H11B	109.5	C7—N2—Zn1	113.8 (5)
C10—C11—H11C	109.5	C13—N3—C18	119.4 (7)
H11A—C11—H11C	109.5	C13—N3—Zn2	126.6 (5)
H11B—C11—H11C	109.5	C18—N3—Zn2	114.0 (6)
N2—C12—C10	123.4 (8)	C19—N4—C24	120.5 (7)
N2—C12—H12	118.3	C19—N4—Zn2	114.0 (5)
C10—C12—H12	118.3	C24—N4—Zn2	125.5 (5)
			
N1—C1—C2—C4	−0.3 (13)	N2—Zn1—N1—C1	−179.1 (6)
N1—C1—C2—C3	177.9 (7)	Cl1—Zn1—N1—C1	−65.2 (7)
C1—C2—C4—C5	0.8 (13)	Cl2—Zn1—N1—C1	71.6 (7)
C3—C2—C4—C5	−177.4 (9)	N2—Zn1—N1—C6	−1.4 (5)
C2—C4—C5—C6	−1.5 (14)	Cl1—Zn1—N1—C6	112.5 (5)
C4—C5—C6—N1	1.7 (12)	Cl2—Zn1—N1—C6	−110.7 (5)
C4—C5—C6—C7	−179.5 (8)	C10—C12—N2—C7	−1.2 (12)
N1—C6—C7—N2	−1.5 (9)	C10—C12—N2—Zn1	178.9 (6)
C5—C6—C7—N2	179.6 (8)	C8—C7—N2—C12	0.8 (12)
N1—C6—C7—C8	178.1 (7)	C6—C7—N2—C12	−179.6 (6)
C5—C6—C7—C8	−0.8 (11)	C8—C7—N2—Zn1	−179.3 (6)
N2—C7—C8—C9	−0.6 (12)	C6—C7—N2—Zn1	0.3 (9)
C6—C7—C8—C9	179.8 (7)	N1—Zn1—N2—C12	−179.6 (7)
C7—C8—C9—C10	0.8 (13)	Cl1—Zn1—N2—C12	70.3 (7)
C8—C9—C10—C12	−1.2 (12)	Cl2—Zn1—N2—C12	−64.0 (7)
C8—C9—C10—C11	178.3 (8)	N1—Zn1—N2—C7	0.5 (6)
C9—C10—C12—N2	1.4 (12)	Cl1—Zn1—N2—C7	−109.6 (5)
C11—C10—C12—N2	−178.1 (8)	Cl2—Zn1—N2—C7	116.2 (6)
N3—C13—C14—C16	−1.8 (12)	C14—C13—N3—C18	−0.1 (12)
N3—C13—C14—C15	179.3 (8)	C14—C13—N3—Zn2	−178.1 (6)
C13—C14—C16—C17	3.1 (13)	C17—C18—N3—C13	0.8 (13)
C15—C14—C16—C17	−178.1 (9)	C19—C18—N3—C13	−178.5 (7)
C14—C16—C17—C18	−2.6 (15)	C17—C18—N3—Zn2	179.0 (7)
C16—C17—C18—N3	0.5 (15)	C19—C18—N3—Zn2	−0.3 (10)
C16—C17—C18—C19	179.8 (8)	N4—Zn2—N3—C13	178.9 (7)
N3—C18—C19—N4	−0.8 (11)	Cl4—Zn2—N3—C13	−70.6 (7)
C17—C18—C19—N4	179.9 (8)	Cl3—Zn2—N3—C13	66.0 (6)
N3—C18—C19—C20	−179.3 (7)	N4—Zn2—N3—C18	0.8 (6)
C17—C18—C19—C20	1.4 (14)	Cl4—Zn2—N3—C18	111.3 (6)
N4—C19—C20—C21	3.6 (12)	Cl3—Zn2—N3—C18	−112.1 (6)
C18—C19—C20—C21	−178.0 (8)	C20—C19—N4—C24	−1.7 (10)
C19—C20—C21—C22	−4.0 (13)	C18—C19—N4—C24	179.7 (7)
C20—C21—C22—C24	2.4 (12)	C20—C19—N4—Zn2	−179.9 (6)
C20—C21—C22—C23	−178.8 (8)	C18—C19—N4—Zn2	1.5 (8)
C21—C22—C24—N4	−0.5 (11)	C22—C24—N4—C19	0.3 (11)
C23—C22—C24—N4	−179.4 (7)	C22—C24—N4—Zn2	178.3 (5)
C2—C1—N1—C6	0.5 (11)	N3—Zn2—N4—C19	−1.3 (5)
C2—C1—N1—Zn1	178.2 (6)	Cl4—Zn2—N4—C19	−113.6 (5)
C5—C6—N1—C1	−1.2 (10)	Cl3—Zn2—N4—C19	108.4 (5)
C7—C6—N1—C1	179.8 (6)	N3—Zn2—N4—C24	−179.4 (6)
C5—C6—N1—Zn1	−179.1 (6)	Cl4—Zn2—N4—C24	68.3 (6)
C7—C6—N1—Zn1	1.9 (7)	Cl3—Zn2—N4—C24	−69.7 (6)

### Hydrogen-bond geometry (Å, °)

**Table:**

*D*—H···*A*	*D*—H	H···*A*	*D*···*A*	*D*—H···*A*
C1—H1···Cl3^i^	0.93	2.82	3.516 (8)	132
C16—H16···Cl3^ii^	0.93	2.83	3.638 (10)	146
C3—H3A···Cg5	0.96	3.10	3.719 (6)	124
C11—H11A···Cg2^iii^	0.96	2.83	3.688 (5)	150
C15—H15C···Cg1^iv^	0.96	2.84	3.704 (6)	150
C23—H23C···Cg4	0.96	3.11	3.690 (6)	120

Symmetry codes: (i) *x*+1/2, −*y*−3/2, *z*; (ii) *x*+1/2, −*y*−5/2, *z*;  (iii) *x*, *y*+1, *z*; (iv) *x*, *y*−1, *z*.
